# Inflammatory Cytokines as Risk Factors for a First Venous Thrombosis: A Prospective Population-Based Study

**DOI:** 10.1371/journal.pmed.0030334

**Published:** 2006-08-22

**Authors:** Sverre C Christiansen, Inger Anne Næss, Suzanne C Cannegieter, Jens Hammerstrøm, Frits R Rosendaal, Pieter H Reitsma

**Affiliations:** 1 Department of Clinical Epidemiology, Leiden University Medical Center, Leiden, Netherlands; 2 Department of Cancer Research and Molecular Medicine, Norwegian University of Science and Technology, Trondheim, Norway; 3 Department of Haematology, Leiden University Medical Center, Leiden, Netherlands; 4 Laboratory for Experimental Internal Medicine, Academic Medical Center, Amsterdam, Netherlands; University of Aberdeen, United Kingdom

## Abstract

**Background:**

In case-control studies, elevated levels of interleukins 6 and 8 have been found to be associated with an increased risk of venous thrombosis (VT). Because of the design of these studies, it remained uncertain whether these alterations were a cause or a result of the VT. In order to distinguish between the two, we set out to measure the levels of six inflammatory markers prior to thrombosis in a population-based cohort using a nested case-cohort design.

**Methods and Findings:**

Between August 1995 and June 1997, blood was collected from 66,140 people in the second Norwegian Health Study of Nord-Trøndelag (HUNT2). We identified venous thrombotic events occurring between entry and 1 January 2002. By this date we had registered 506 cases with a first VT; an age- and sex-stratified random sample of 1,464 controls without previous VT was drawn from the original cohort. Levels of interleukins 1β, 6, 8, 10, 12p70, and tumour necrosis factor-α were measured in the baseline sample that was taken 2 d to 75 mo before the event (median 33 mo). Cut-off points for levels were the 80th, 90th, and 95th percentile in the control group. With odds ratios ranging from 0.9 (95% CI: 0.6–1.5) to 1.1 (95% CI: 0.7–1.8), we did not find evidence for a relationship between VT and an altered inflammatory profile.

**Conclusions:**

The results from this population sample show that an altered inflammatory profile is more likely to be a result rather than a cause of VT, although short-term effects of transiently elevated levels cannot be ruled out.

## Abstract 2

An altered inflammatory profile appears more likely to be a result than a cause of venous thrombosis.

## Editors' Summary

**Background.**

Blood clots (thromboses) are a common medical problem, especially in people who have been immobilized for a variety of reasons, who have other medical or surgical conditions, or who take certain types of drugs such as oral contraceptives. As well as these factors, various genetic changes make it more likely that certain people will develop a thrombosis. The most well known of these genetic changes is in a clotting factor, Factor V. Instead of the normal clotting factor V, some people have a variant known as Factor V Leiden, which makes them more likely to develop a thrombosis. (It is so named as it was discovered by researchers in Leiden, Netherlands.) Researchers are trying to find out if other abnormalities, particularly in levels of substances involved in inflammation, might make the chance of thrombosis more common. Identifying such changes might make it easier to predict who might be at risk of getting a thrombosis—for example, when a patient has to have an operation—and thus give them appropriate preventative measures.

**Why Was This Study Done?**

Previous studies have shown that the levels of inflammatory substances, known as cytokines, are raised around the time of a thrombosis. However, because of the design of these studies it was not clear whether these alterations were a cause or a result of the thrombosis. These researchers wanted to measure the levels of six cytokines before thrombosis in a large group of people and follow them to see if any particular level of cytokine made it more likely that they would go on to develop a thrombosis.

**What Did the Researchers Do and Find?**

During a three-year period between August 1995 and June 1997, blood was collected from 66,140 people in the second Norwegian Health Study of Nord-Trøndelag (HUNT2). Anyone who had a thrombosis between the start of the study and the beginning of 2002 was identified—506 people in all. Blood samples, taken from these people (cases) between two days and 75 months before the thrombosis happened, were used to measure levels of a number of different cytokines (abbreviated to IL-1β, IL-6, IL-8, IL-10, IL12p70, and tumour necrosis factor alpha [TNF-α]). These levels were then compared with those in samples also taken earlier from 1,464 people who were similar to the cases but had no thrombosis (controls). The authors found no evidence for a relationship between the chance of getting a thrombosis and a change in any of these markers of inflammation.

**What Do These Findings Mean?**

It seems unlikely that any long-term changes in levels of cytokines make any difference as to whether or not people develop a thrombosis. Hence, any changes seen in previous studies are most likely to have been the result of the thrombosis. However, the researchers could not rule out that changes occurred in the hours or days immediately before the thrombosis, although that seems unlikely. In any case, the levels of these cytokines do not seem to be useful as a clinical tool to predict who is at risk of thrombosis.

**Additional Information.**

Please access these Web sites via the online version of this summary at http://dx.doi.org/101371/journal.pmed.0030334.

- MedlinePlus encyclopedia entries on deep venous thrombosis and pulmonary embolus
- Omni, a health information service in the UK run by the Resource Discovery Network, has links to pages of information on venous thrombosis
- The HUNT studies are described in this Web site

## Introduction

Venous thrombosis (VT) is a common and potentially lethal disorder with an estimated incidence of one to three per 1,000 individuals per year [1–3]. The occurrence of venous thrombotic events is influenced by acquired and inherited risk factors (e.g., oral contraception and Factor V Leiden) [4,5]. Combinations of these risk factors further increase the risk to potentially high levels [6].

An important subgroup of risk factors comprises the levels of procoagulant proteins. Several studies have shown that elevated levels of coagulation factors VIII, IX, and XI increase the thrombotic risk [7–9]. Genetic determinants of these high levels have not been found, with the exception of the well-known relation between the ABO blood group and factor VIII levels. It is also possible that increased procoagulant levels are acquired. Experimental studies in human volunteers injected with low-dose endotoxin provide credence for this possibility, as they showed increases in procoagulant protein levels in parallel with an inflammatory response [10–14]. In other studies we have found increased levels of inflammatory markers in patients who had suffered from venous thrombotic disease [15–17]. A scenario that links all these data together is that an acquired inflammatory component is either wholly or in part responsible for the increased levels of at least some of the procoagulant proteins, and, either directly or through this mechanism, causes VT.

The association between inflammation and VT leads to the question of whether inflammation is the cause or the result of VT. There are arguments for both. In favour of a causative role is the above-mentioned possibility that inflammation may increase procoagulant protein levels and thus increase the prothrombotic state of the blood. In addition, it is well known that inflammation may promote tissue factor expression on white blood cells and endothelial cells, thus providing a trigger that may lead to thrombotic disease [18–22]. Other arguments that could go with either role are: 1) the fact that levels of inflammation markers are high in the acute phase of VT and come down afterwards [23], and 2) the frequent occurrence of inflammatory post-thrombotic syndrome [24,25].

Recent case-control studies showed that raised levels of interleukins (IL) 6 and 8 are associated with a 2-fold risk for both first and recurrent thromboembolic events [15,16]. In the Leiden Thrombophilia Study (LETS), both cytokines and tumour necrosis factor alpha (TNF-α) were associated with a 2- to 3-fold increased risk of a first VT, whereas the risk for IL-10 seemed to be decreased [17]. These studies don't answer the question of whether inflammation is a cause or a result of the event. No prospective studies have yet been undertaken to find out whether increased cytokine levels can be demonstrated in the blood before a venous thromboembolic event has occurred.

We tested the hypothesis that a chronic inflammatory state following a proinflammatory stimulus, regardless of origin, could precede future thrombotic events. In a population-based cohort, we tested levels of cytokines (TNF-α, IL-1β, IL-6, IL-8, IL-12p70) in blood samples obtained at inclusion, and examined the association with the occurrence of subsequent thrombosis.

## Methods

### Patients and Controls

Between August 1995 and June 1997, all inhabitants aged 20 y or older (*n =* 94,194) in Nord-Trøndelag County (in Middle Norway) were invited to participate in a population-based health survey (HUNT2) [26]. HUNT2 covers a wide range of topics such as chronic diseases, mental diseases, medication, education, employment, physical activity, and quality of life. The population in this county is considered representative of the rest of the Norwegian population regarding age and sex distribution, morbidity, mortality, and income. It has a low geographic mobility, which makes it suitable for a population survey with subsequent follow-up.

The overall participation rate was 71% (*n =* 66,140) at baseline (1995–1997), with a median age of 46 y (range 19–103 y) [26].

All consenting participants (*n =* 66,140) underwent a physical examination and filled out a questionnaire at inclusion. The questionnaire contained questions about risk factors for cardiovascular disease, lifestyle, quality of life, and medication use. In addition, all participants were invited to donate 7.5 ml of blood, and acceptable serum samples were obtained from 65,291 participants (98.7%) [26].

The follow-up for VT was performed as follows: the computerized diagnosis registries of all departments of the only two local hospitals (Levanger and Namsos hospitals) were checked for all in- and outpatient diagnoses containing ICD-9 and ICD-10 diagnostic codes for VT up until 1 January 2002. We completed case finding by searching positive diagnostic procedure codes for venography, duplex ultrasound, and Doppler ultrasound from the registries of the radiology departments in the two hospitals, and patients from Nord-Trøndelag County discharged from St. Olav University Hospital in the neighbouring county with diagnostic codes of VT. This search led to the identification of 2,136 cases with a diagnostic code of VT. Hospital records were obtained, and two physicians (I. Næss and S. Christiansen) validated each case. Cases were included only if they fulfilled the following criteria: deep venous thrombosis (DVT), an intraluminal filling defect or no venous filling on ascending contrast venography; or, no compressible venous segment or no venous flow in popliteal, femoral, or axillar veins on duplex ultrasound; or, a positive computed tomography scan or a positive autopsy; for pulmonary embolus (PE), a ventilation-perfusion scan with one or more segmental or subsegmental perfusion defects with normal ventilation; or, a contrast defect on pulmonary computed tomography scan; or, a positive autopsy. This way, 1,271 cases with a validated diagnosis of definite and probable VT were identified. These records were linked to the HUNT2 cohort, and 798 (63%) cases were identified within the cohort. We excluded all patients with previous VT, enrolment in the HUNT2 cohort after the event, or eye-vein thrombosis (*n =* 283). As a consequence, 515 cases were included, out of which 506 had blood samples available in which reliable laboratory tests could be performed.

1,505 controls were randomly sampled from the baseline of the HUNT2 cohort by frequency matching to the cases by sex and 5-y age strata. 29 of them were excluded from the subsequent data analysis because they had suffered one or more thrombotic events before the drawing of a blood sample. Another 12 had missing blood samples, leaving 1,464 controls for the analyses.

In a case-cohort design, every person in the source population has the same chance of being included as a control. Since our control group was sampled at baseline, 29 controls later became cases during the follow-up period. The odds ratio in a case-cohort study is to be interpreted as an estimate of the risk ratio, because all cohort participants from the baseline contribute to the denominator in absolute risks [27]. As a consequence of this design, the 29 controls that later became cases were included in the case group as well as in the control group.

### Laboratory Measurements

Whole-blood samples were collected from non-fasting participants at entry in the HUNT2 cohort, and within 2 h the serum was separated from blood cells (centrifugation at 1,010 × g) and stored in a refrigerator at 4 °C. All samples were transported the same day in a cooler to the central laboratory at Levanger Hospital and stored in the HUNT biobank there the next morning at −70 °C. Samples taken on Friday were frozen the following Monday morning after being transported the same Friday evening [26]. We have no indications that the time between phlebotomy and freezing affected the levels of the inflammatory mediators.

Serum levels of IL-1β, IL-6, IL-8, IL-12p70, and TNF-α were measured using a commercial multiplex bead assay that allows accurate and reproducible measuring of multiple cytokines in a single small serum aliquot [28,29]. To lower the detection limit of the kit, we used procedures that differed slightly from the recommendations of the manufacturer (BD Biosciences, Alphen aan den Rijn, Netherlands). In brief, 10 μl of thawed serum was mixed with 10 μl of diluted capture bead suspension and 10 μl of phycoerythrin-labelled detection reagent in a sample tube [17]. After 3 h of incubation in darkness, 200 μl of wash buffer was added and the samples were centrifuged at 200 × g. The supernatant was aspirated and the bead pellet resuspended in 150 μl of wash buffer, followed by analysis on a FACSCalibur flow cytometer. (BD Biosciences) [17]. In our hands, the detection limit of these ELISAs was 2.5 pg/ml for all cytokines. With the bead assays, the cytokine concentrations were often near the concentration of the lowest calibrator, and, depending on the analyte, the levels of cytokine in part of the samples remained undetectable. The technicians were not aware of the case or control status of the samples.

In some samples, outlier levels far above the standard curves (up to 4% in cases and 3% in controls) were seen. We believe that these measurements represent artefacts of the procedure, and the results from such samples were excluded prior to the analysis. In 12 controls (0.8%) and nine cases (1.7%), the samples were missing, the volume of serum was too low to perform cytokine assays, or the cytokine measurements failed for technical reasons.

### Statistical Analysis

We used univariate logistic regression to calculate odds ratios and their confidence intervals. Cut-off points were detectable cytokine levels at the 80th, 90th, or 95th percentile calculated in control participants. We stratified for sex, three age categories (<50, 50–69, and ≥70 y of age), time between blood sampling and the event, and whether the event was idiopathic or secondary. A secondary event was defined as an event that occurred after major surgery or trauma, during marked immobility (specified as paresis, paralysis, or >8 h of travel), ante- or postpartum, during use of oral contraceptive pills, or in people with a history of malignancy. Linear regression was used to evaluate the relation between cytokine levels and time until the events in cases.

All analyses were performed with SPSS version 11.0 (SPSS, Chicago, Illinois, United States).

### Consent

Participation was voluntary, and each participant signed a written consent. All surveys were approved by the Norwegian Data Inspectorate and by the Regional Committee for Ethics in Medical Research.

## Results

General characteristics of the patients and controls are listed in Table 1. The median age was 70 y in both cases and controls at baseline. 45.1% of the cases were men, compared with 46% in the control group (Table 1). Table 2 summarizes the cytokine and chemokine data. TNF-α, IL-6, and IL-10 were detectable in approximately two-fifths of the samples, whereas IL-1β and IL-12p70 were detectable in three-fifths of the samples. IL-8 was unusual in that it was detectable in almost all cases (99%) and in almost all the controls (98%) as well (Table 2).

**Table 1:**
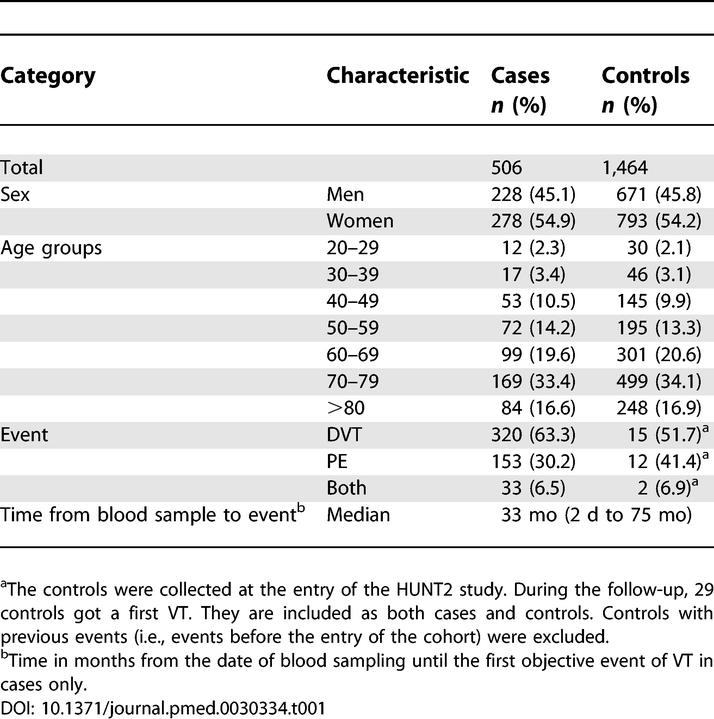
General Characteristics of Cases and Controls

**Table 2:**
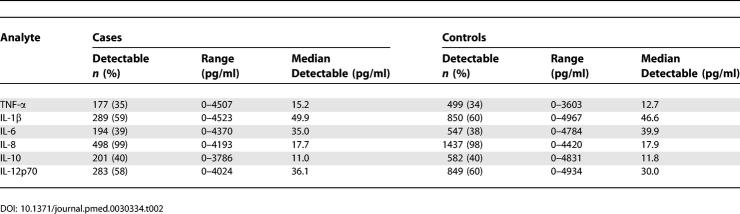
Levels of Inflammatory Markers in Cases and Controls

Whether markers were detectable or not was similar for cases and controls. Consequently, the odds ratios calculated based on detectable versus undetectable levels were about 1.0, with narrow confidence limits (Table 3). This indicates that a detectable level of any of the markers surveyed in this study was not related to a subsequent VT.

**Table 3:**
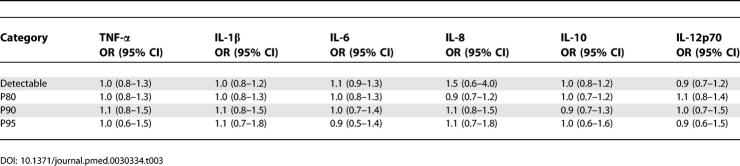
Odds Ratios and 95% Confidence Intervals for Detectability, and Cut-Offs at the 80th, 90th, and 95th Percentile of Inflammatory Markers

Table 3 lists the odds ratios that were calculated using P80, P90, and P95 as cut-offs. All interleukins demonstrated the same pattern, with no odds ratios exceeding 1.1 for the proinflammatory interleukins (TNF-α, IL-1β, IL-6, IL-8, and IL-12p70) and an odds ratio of 0.9 for the anti-inflammatory IL-10.

**Figure 1:**
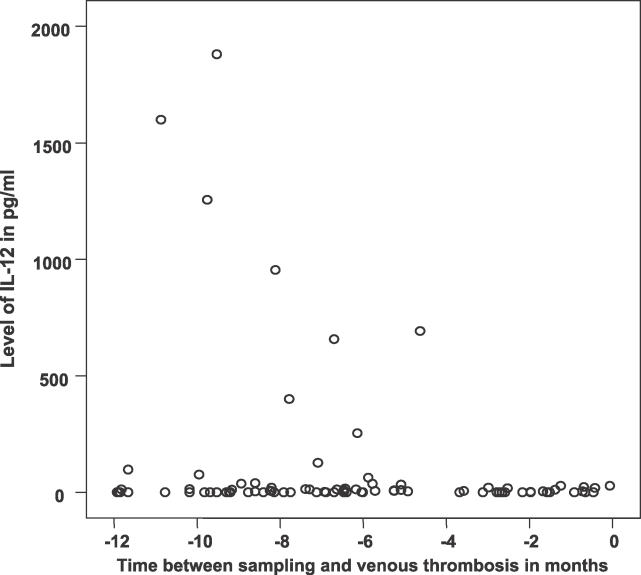
Regression Analysis of the Levels of IL-12 in the 12-mo Period before a Thrombotic Event (*p* = 0.04) The *y*-axis shows levels of IL-12 from 85 individuals. The *x*-axis shows IL-12 levels for those individuals for whom data were available between sampling and thrombotic event. Note that IL-12 levels do not show a tendency to be increased when they are measured closer to the thrombotic event; in fact, the opposite appears to be the case.

We performed a sub-analysis on the samples that were taken within 1 y before the event to see if levels were higher shortly before a thrombosis occurred. They were not; if anything, they tended to be lower. In an analysis of only cases, IL-12 levels, for instance, showed a significant decrease with time closer to the event (Figure 1). IL-1 showed a very similar pattern, while the other cytokines showed no clear trends in values nearer to the event.

Using the 90th percentile in the control participants as a cut-off point for all markers, again no effect was found when men and women were assessed separately, or when a distinction was made between an idiopathic and a secondary event. We found no effect within the three age categories, either (Table 4).

**Table 4:**
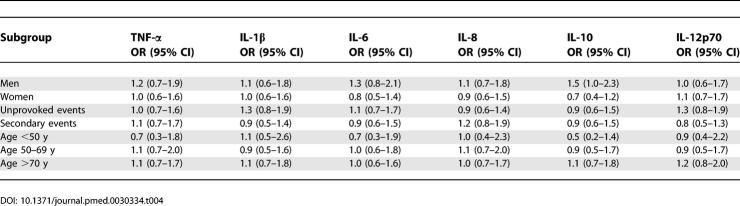
Odds Ratios and 95% Confidence Intervals for Subgroups with Inflammatory Markers above the 90th Percentile Compared with Those below the 90th Percentile

## Discussion

We performed the current study to test the hypothesis that circulating levels of proinflammatory cytokines or chemokines are associated with a future event of VT. The data negate this hypothesis, as we found no evidence, neither in a primary analysis nor in post-hoc sub-analyses, that levels of inflammation markers were increased in individuals who later developed VT.

Due to the prospective design of this case-cohort study, there was considerable variation in the time between the blood sampling and the event (this varied between 75 mo and 2 d). Therefore, we could not fully rule out the possibility of a sudden increase of inflammatory markers in hours or days before a first thrombosis. However, this possibility seems less likely, considering the results of a sub-analysis of cases developing VT in the first year after blood sampling. These patients showed no increase whatsoever in cytokine levels shortly before the event.

We found a higher prevalence of detectable cytokines as compared with the prevalence recently found in the control group in the LETS, although the median levels were comparable [17]. Both our cases and controls (with a mean and median age of 66 y and 70 y, respectively) (Table 1) could be described as a substantially older population than the LETS control group (mean age 47 y) [17]. As some cytokines have been shown to increase with age while others remain constant, it is possible that the higher prevalence could be partly due to the high age of our cohort [30,31]. This does not, however, explain the discrepancy of our study with the increased risks of VT recently found in the LETS.

The LETS found that the risk of VT increased parallel to several cytokines (TNF-α, IL-6, and IL-8). This was supported by a decreased risk with increasing levels of anti-inflammatory IL-10. In the LETS, the association between cytokine levels and VT was found in samples that were collected after the event. Therefore, a model in which the thrombosis is the cause of subsequent cytokine release, and not the other way around, is the most likely explanation of the results. Such a model is also supported by the fact that C-reactive protein levels were not associated with VT in the LITE prospective study [32].

A potential limitation of the study is that the inflammatory markers were evaluated with a method with lower sensitivity than high-sensitivity ELISAs. For example, a high-sensitivity ELISA of IL-6 may yield detectable levels in more than 90% of the control samples. On the other hand, the claims that inflammatory markers are a risk factor for VT were based on studies of similar size with assays similar to ours.

In conclusion, we have investigated the role of cytokines before development of a first VT in the general population, and found no evidence of an association, not even shortly before the event. Therefore, it is unlikely that cytokine levels play an important role in determining the risk of VT.

## Abbreviations

DVT: deep venous thrombosis
HUNT2: second Norwegian Health Study of Nord-Trøndelag
IL: interleukin
LETS: Leiden Thrombophilia Study
PE: pulmonary embolus
TNF-α: tumour necrosis factor alpha
VT: venous thrombosis
